# A Digital Health Initiative (COVIDsmart) for Remote Data Collection and Study of COVID-19’s Impact on the State of Virginia: Prospective Cohort Study

**DOI:** 10.2196/37550

**Published:** 2023-03-15

**Authors:** Josh Schilling, Dave Klein, Marilyn M Bartholmae, Sepideh Shokouhi, Angela J Toepp, Amira A Roess, Joshua M Sill, Matvey V Karpov, Kathleen Maney, K Pearson Brown, Brian L Levy, Keith D Renshaw, Sunita Dodani, Praduman Jain

**Affiliations:** 1 Vibrent Health Fairfax, VA United States; 2 Department of Psychiatry and Behavioral Health Eastern Virginia Medical School Norfolk, VA United States; 3 Sentara Healthcare Analytics and Delivery Science Institute Eastern Virginia Medical School Norfolk, VA United States; 4 Sentara Healthcare System Norfolk, VA United States; 5 George Mason University Fairfax, VA United States; 6 Department of Internal Medicine Eastern Virginia Medical School Norfolk, VA United States

**Keywords:** COVID-19, digital health technology, human subjects, partnership, community health, diversity, mobile health, mHealth, medical subject headings, MeSH, medical informatics, internet, digital health, digital solution, digital recruitment, precision medicine, digital marketing, decision-making, COVIDsmart

## Abstract

**Background:**

The COVID-19 pandemic has affected people's lives beyond severe and long-term physical health symptoms. Social distancing and quarantine have led to adverse mental health outcomes. COVID-19–induced economic setbacks have also likely exacerbated the psychological distress affecting broader aspects of physical and mental well-being. Remote digital health studies can provide information about the pandemic's socioeconomic, mental, and physical impact. COVIDsmart was a collaborative effort to deploy a complex digital health research study to understand the impact of the pandemic on diverse populations. We describe how digital tools were used to capture the effects of the pandemic on the overall well-being of diverse communities across large geographical areas within the state of Virginia.

**Objective:**

The aim is to describe the digital recruitment strategies and data collection tools applied in the COVIDsmart study and share the preliminary study results.

**Methods:**

COVIDsmart conducted digital recruitment, e-Consent, and survey collection through a Health Insurance Portability and Accountability Act–compliant digital health platform. This is an alternative to the traditional in-person recruitment and onboarding method used for studies. Participants in Virginia were actively recruited over 3 months using widespread digital marketing strategies. Six months of data were collected remotely on participant demographics, COVID-19 clinical parameters, health perceptions, mental and physical health, resilience, vaccination status, education or work functioning, social or family functioning, and economic impact. Data were collected using validated questionnaires or surveys, completed in a cyclical fashion and reviewed by an expert panel. To retain a high level of engagement throughout the study, participants were incentivized to stay enrolled and complete more surveys to further their chances of receiving a monthly gift card and one of multiple grand prizes.

**Results:**

Virtual recruitment demonstrated relatively high rates of interest in Virginia (N=3737), and 782 (21.1%) consented to participate in the study. The most successful recruitment technique was the effective use of newsletters or emails (n=326, 41.7%). The primary reason for contributing as a study participant was advancing research (n=625, 79.9%), followed by the need to give back to their community (n=507, 64.8%). Incentives were only reported as a reason among 21% (n=164) of the consented participants. Overall, the primary reason for contributing as a study participant was attributed to altruism at 88.6% (n=693).

**Conclusions:**

The COVID-19 pandemic has accelerated the need for digital transformation in research. COVIDsmart is a statewide prospective cohort to study the impact of COVID-19 on Virginians' social, physical, and mental health. The study design, project management, and collaborative efforts led to the development of effective digital recruitment, enrollment, and data collection strategies to evaluate the pandemic’s effects on a large, diverse population. These findings may inform effective recruitment techniques across diverse communities and participants' interest in remote digital health studies.

## Introduction

The COVID-19 pandemic reached the United States with little warning and significantly disrupted the lives of nearly all individuals. A COVID-19 crisis gripped our nation in March 2020, continued into 2021 with 2 waves of the outbreak, leading to enormous stress, triggering an exacerbation of mental illness and substance use disorders [1,2]. As the current omicron variant–led third wave of cases surges worldwide, we are faced with more questions and uncertainty than during the first 2 waves. The resurgence of the virus is a huge setback for the countries that had largely succeeded in bringing infection rates down to manageable levels after implementing drastic lockdowns [3]. The upside of the present situation is that as more tests become available and more people are vaccinated, people are less likely to be hospitalized and die from the virus [4,5].

Since its emergence in 2019, COVID-19 has impacted people’s lives in multifaceted ways. The SARS-CoV-2 infection can result in critical illness and mortality in some patients, including individuals with chronic diseases and immunodeficiencies [6]. Recent observations show evidence of post–COVID-19 condition, defined as a broad range of symptoms that can remain weeks after the clearance of the acute infection [7]. Long-term lockdowns, social distancing, and reduced community engagement activities have increased psychological distress and mental vulnerability to anxiety and depression [1]. The COVID-19–induced shutdown of economic activities has also led to unique social, environmental, and economical changes that continue to impact people’s lives in unprecedented ways. In the United States, demographic factors, such as race, age, gender, income, and education, have affected vulnerability to the COVID-19 pandemic [8]. There is growing evidence of disparities between different population groups in economic hardship, mental health symptoms, and mortality due to COVID-19 [9]. The sudden change from normal life to an era of quarantine has also disrupted the system of providing health care, further exacerbating disparities. People are spending more time than ever living and working in the digital milieu propelling virtual platforms to an unprecedented level [10]. Since the onset of the pandemic, medical and laboratory professionals have completely modified the organization of their work and their relationship with patients. In an attempt to limit the community spread of COVID-19, health care providers tried to transition into virtual care despite the lack of an established infrastructure. This has resulted in delays in care where both physicians and patients are learning to navigate the telemedicine system. This change in the health care environment, in-person to virtual, has left many patients feeling they are not receiving the same quality of care [11].

The COVID-19 pandemic also had an effect on research by greatly limiting the ability of researchers to collect data in person. Yet, this was when data surrounding the impact of the pandemic were most valuable. Issues arose around how to safely administer the study and the materials, without placing the participant and research staff at risk of infection. This limitation to in-person research techniques has forced researchers to switch to digital health research platforms [10].

While advances in digital health technologies have improved research studies and created opportunities for outreach, the COVID-19 pandemic has created an unprecedented level of disruption on research and how relevant data can be collected securely within the Health Insurance Portability and Accountability Act (HIPAA) guidelines [12]. By forging a partnership, institutional strengths can be leveraged when mitigating the risks and challenges in recruiting participants for remote studies in health research, as poor recruitment could diminish the scientific value of a study.

Digital health studies have enabled scientists to obtain data from various sources across large geographic areas and diverse populations. This rapid advancement in the use of technology has allowed research to continue through many situations when operational efforts are costly or impractical, as may be the case when at-home stay may be required of participants [13]. When implemented effectively, digital cohorts can also help us better understand inequities in the impact of COVID-19 and reduce health disparities by broadening our reach into communities. Digital health technology and multi-institutional partnerships may be used to improve the representation of diverse demographic groups by increasing access to studies and the retention of participants while lowering operational overhead in managing large studies.

The state of Virginia's demographic data show evidence of a growing rural-urban divide and distinct socioeconomic differences between the multiethnic population of Northern Virginia and the predominately white population in rural communities. This unique sociodemographic distribution offers an interesting and important source to study the multifaceted impact of the COVID-19 pandemic in a broad range of lifestyle, environmental, economic, and social settings. Vibrent Health, Eastern Virginia Medical School (EVMS)–Sentara Healthcare Analytics and Delivery Science Institute (HADSI), and George Mason University collaboratively designed and deployed a statewide digital health research study called COVIDsmart to understand the effects of COVID-19 on the mental health and well-being of communities across large geographical areas. Therefore, this paper aims to describe the digital solutions applied in implementing the COVIDsmart study. Furthermore, this paper discusses the successful use of digital health technology and multi-institutional partnership in COVIDsmart to recruit participants from various rural and urban communities and assess the primary motivations for study participation.

## Methods

### COVIDsmart Study

COVIDsmart is a statewide prospective cohort study focused on understanding the impact of COVID-19 on the social, physical, and mental health of Virginians. The study is a collaborative effort across multi-institutional and multidisciplinary groups from EVMS-Sentara HADSI, Vibrent Health, and George Mason University.

### Ethics Approval

The research protocol and validated survey instruments were created jointly and reviewed by an expert panel. The protocol was approved by the EVMS Institutional Review Board (20-07-EX-0138).

### Setting and Study Participant Reach

COVIDsmart is currently collecting 6-month follow-up data on recruited participants in Virginia. Specifically, Virginia is a unique state with a more than 8.5 million population across nearly 40,000 square miles. The population covers large urban coastal areas near Washington DC and the Hampton Roads near North Carolina, and a significant rural region along the Appalachian Mountains. Hampton Roads is the Southeastern Coastal region of Virginia, which covers the municipalities of Norfolk, Suffolk, Virginia Beach, Portsmouth, Chesapeake, Tangier Island (part of Chesapeake Bay), Hampton, Williamsburg, Newport News, and some of the surrounding counties of North Carolina. Given the impact of the COVID-19 pandemic on every aspect of daily life, the COVIDsmart study focuses on determining individuals' social, mental, and physical well-being across a diverse state in the United States. Considering the diversity in the state, the population of Virginia is 19.9% Black or African American (13.4% across the United States), 9.8% Hispanic or Latino (18.5% across the United States), and 6.9% Asian (5.9% across the United States) per Census data [14,15]. The impact of this study will direct intervention methods in local communities and across the state.

While the technology component may be considered less of a barrier, there are still concerns in the digital divide. These concerns include the ease and convenience of technology across demographics such as age and education, as well as the digital inequities due to limited cellular data plans and high-speed internet availability outside of institutional access. A report on Virginia’s digital divide in education revealed that Black and Latino student households are more than 2 times more likely to lack a computer or laptop compared to White student households. In addition, among lower-income populations, about 16% lack internet in the home, compared to 13% for middle-income and 8% for high-income populations [4,16]. These hard-to-reach populations were disproportionately impacted by the pandemic, so partnering with practitioners and organizations that have regular contact and provide care for these people was expected to be useful in recruiting a diverse sample.

### Vibrent Health’s Digital Health Solutions Platform

COVIDsmart delivered digital recruitment, e-Consent, a survey design through Vibrent Health’s validated digital health solutions platform (DHSP) as an alternative to traditional in-person recruitment and onboarding [17]. A multi-institutional group of clinical epidemiologists, technology strategists, developers, health service researchers, and behavioral scientists collaborated to better address the complexities of collecting data across different research domains, including physiological, neurological, social neuroscience, socio-psychological, and behavioral economics. This allowed academics from the multi-institutional group, who often lack experience using similar platforms, to use DHSP to design flexible, validated data collection tools and integrate incentives, reminders, and other retention strategies for participation.

**Figure 1:**
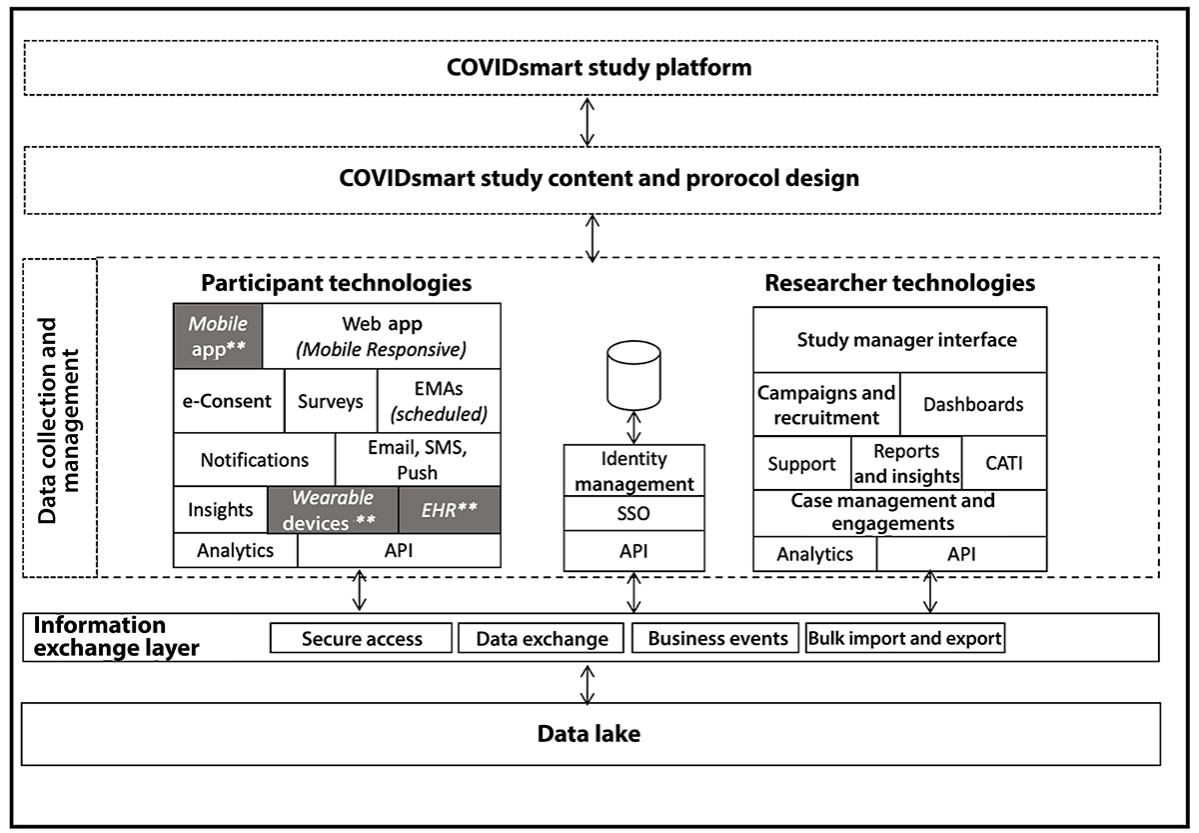
COVIDsmart study platform using Vibrent Health’s digital health solutions platform (DHSP) for the technology infrastructure and research study content and protocol. API: application programming interface; CATI: Computer-assisted Telephone Interview; EHR: electronic health record; EMA: Ecological Momentary Assessment; SSO: Single Sign-On. ** These Vibrent DHSP features were considered but not delivered to participants.

The DHSP managed the collection of study data and secure data transfer to HADSI for analysis and reporting. The platform is supported by advanced security technologies, including HIPAA compliance and secure exchange and storage of study participant data (Figure 1).

### Features of the DHSP Developed for Diverse Participant Use

COVIDsmart enabled cross-platform availability on various smartphones, browsers, and across multiple networks with cloud computing to support low-cost web-connected devices and low network demand. Our goal was to develop a *user-friendly* platform for individuals of all ages, education levels, and technographic profiles. Emails, SMS (short message services), and push notifications were sent to participants to drive campaigns and return rates to the platform to complete ongoing study requests. Participants were informed about the data collection and the comparative insights among other participants as a community-focused information. Data security was based on a HIPAA-compliant cloud infrastructure that included standard protocols and additional security protocols across the Federal Information Security Modernization Act, the Federal Risk and Authorization Management Program, and statements on standards for attestation engagements. Technical support utilities and functions were in place to support questions that arise from participants and manage ticket-based responses to the queries and resolutions.

### Potential for Continued Long-term Engagement and Adaptability

The interactive platform grew to support additional data, new substudies, and flexibility to change with an adaptive research protocol to support the latest needs for treatment and course changes in diseases and outbreaks. The research team shared access to knowledge and participant content or resources for participant support. Resources on the platform can be updated with changing information and support a trusted shared content delivery center for participants to engage and learn using a built-in survey and content designer. Availability and access to the research team’s contact information were provided in case questions arise while supporting changing research personnel when considering a long-term longitudinal study.

A series of study design virtual meetings and collaborative sessions on recruitment and engagement were held, along with selecting which technology components of the DHSP for the comprehensive research occurred throughout the study. Initially, these virtual, web-based workshops occurred frequently to determine the scope and scale of the study design along with the alignment, commitment, and shared goals of the multi-institutional partnership involvement. The ability for the partnership and the COVIDsmart platform’s ability to change in research resulted in a successful launch of the COVIDsmart study. The flexible nature of the COVIDsmart platform led to a successful launch even with changing partnerships and research strategies.

### Digital Recruitment Methods

COVIDsmart used multiple validated strategies for participant recruitment. Digitally focused recruitment relied heavily on replacing in-person recruitment strategies with tailored virtual and media-based opportunities to increase reach through radio, television, email, newsletters, local community networks, social media, and regional forums [18,19]. Each required a level of effort, cost in terms of time, or institutional review board approvals. Overall, the participants primarily learned about the study from the recruitment website after being directed by one of the varying strategies described above.

### Engagement-Based Incentives

Gift card drawings were made based on the participants' enrollment and level of engagement. Participants are incentivized to stay enrolled and complete follow-up surveys to further their chances of receiving a monthly gift card drawing and one of the multiple grand prizes.

### Inclusion Criteria

Individuals who were residents of Virginia, could read English, and were aged 18 years or older qualified to be included in this study. A laptop, mobile device, or web-connected computer was the minimal technology required to access the platform. No additional software or hardware requirements were necessary.

### Survey Instruments

The study was designed to use easy-to-understand surveys in several modules that were specifically designed for the target populations. Survey modules facilitated participant-led, self-paced completion in which participants can fill out one survey and return days later and complete a second survey. Survey data collection occurred at cyclical intervals, where some surveys were repeated every 2 weeks and others every 4 weeks. Completion of baseline questions drove concurrent behavior where subsequent survey modules were not available until initial survey completion. Participants were also allowed to skip some modules that they deemed less important or not applicable to their current situation, such as occupational exposure, as not all participants would have been impacted in that area. The questions added were based on literature (Table 1) and previous survey instrument designs and encapsulated some well-known validated instruments as a method to observe changing behavior risks, such as the Patient Health Questionnaire-9 and the Generalized Anxiety Disorder-7 [20,21].

A mixed methods approach of digital and in-person communication strategies was used to recruit participants aged 18 years and older living in Virginia, focusing on underrepresented populations in research. Various forms of marketing content were developed for dissemination directly to Virginia residents and organizations that could reach target demographics.

**Table 1:** COVIDsmart data collection, survey schedule, and source.

Theme and schedule			Content		Source
**About You (one time)**					
	Demographics	Age, gender, race, ethnicity, marital status, highest level of education, political affiliation, income, health insurance, and veteran		[22,23]	
	Housing characteristics	Residence details—property type, number of total people in residence, number of elderly individuals, and number of children		Expert panel	
	Health literacy	Confidence filling out forms and help with reading materials		Expert panel	
	Health conditions	Diagnosed conditions, family history, vaccination history, and BMI		Expert panel	
	Lifestyle	Tobacco use, alcohol use, and recent travel		Expert panel	
**COVID Pulse (every** **2 weeks)**					
	Exposure status	Current status, symptoms, testing, willing to get tested, and reason for testing		CDC^a^ Human Infection with 2019 Novel Coronavirus Case Report Form [24]	
	Exposure risk	Exposure to others, close contact status, and close contact symptoms		Expert panel	
	Lifestyle impact	Physical activity, social media, social behavior, alcohol consumption, tobacco usage, cannabis use, mask usage, and public shopping		[25]	
	Community impact	Impact and impact duration expectations		Expert panel	
	Vaccine	Status and reasons not to vaccinate		[26,27]	
**Mood (every 4 weeks)**					
	Social network	Social network index and loneliness index		[21,28,29]	
	Depression	PHQ-9^b^		[21]	
	Anxiety	GAD-7^c^		[20]	
	Financial	Current income status or change, financial performance, and judgement		Expert panel	
**Occupational Exposure (every 4 weeks)**					
	Employment	Status, employment category, essential worker status, and medical occupation (if applicable)		[30]	
	Employment risks	Workplace conditions, safety materials, use of safety materials; contact exposure, type, and duration; and close contacts’ occupation risk		Expert panel	

^a^CDC: Centers for Disease Control and Prevention.

^b^PHQ-9: Patient Health Questionnaire-9.

^c^GAD-7: Generalized Anxiety Disorder-7.

### Outcome Measures

The primary outcome parameters were the ability to launch, recruit, and engage participants for ongoing data collection and research opportunities in developing intervention and public health awareness of the COVID-19 pandemic in Virginia.

## Results

Preliminary results are measured across the convergence of the participants arriving at the recruitment site through to completion of study e-Consent (ie, study enrollment). The recruitment and enrollment results are summarized in Table 2. Almost half of the people who visited the recruitment site were on their mobile devices (n=2147, 44.0%), with the majority of them on an Apple iPhone (n=2686, 55.0%). From the state of Virginia, 3737 users visited the website, and 782 consented to participate in the study at a conversion rate of 20.9%. The most successful recruitment modality resulting in study enrollment was the use of newsletters or emails that popularized the study across the target catchment areas within Virginia.

**Table 2:** Recruitment and participation results (March 1 to May 31, 2021).

Recruitment, participation, and medium		Participants (N=4883), n (%)
**People visiting recruitment site**		
	Direct links	3662 (75.0)
	Social media (Facebook)	229 (4.7)
	Mobile users	2147 (44.0)
	iPhone users	2686 (55.0)
	United States	4847 (99.3)
	Virginia users	3737 (76.5)
**People visiting registration site**		
	Convergence from recruitment site (Virginia users)	1644 (44.0)
**Registration, not yet consented**		
	Convergence from registration site (Virginia users)	950 (57.8)
**Consented participants**		
	Convergence from registered users	782 (82.3)
	Overall convergence (Virginia users)	782 (20.9)
**Recruitment methods (consented participants)**		
	Newsletters or emails	326 (41.7)
	Television advertisements	79 (10.1)
	News publications	131 (16.8)
	Social media	62 (7.9)
**Participation reasons (consented participants)**		
	Advancing research	623 (79.7)
	Giving back to their community	507 (64.8)
	Incentives	164 (21.0)
	Knowledge about myself	120 (15.4)
	Content and information access	99 (12.6)

The most commonly reported reason for participation, or the reason why participants signed up, was to help advance research or give back to the community, accounting for 88.6% (n=693) of participant responses.

With regards to the geographic distribution, we were able to recruit participants from 55 counties and 183 zip codes across the state of Virginia. Participants from both high-density urban counties (eg, Fairfax and Prince William) and rural counties (eg, Mecklenburg, Louisa, and Buchanan) with predominately White populations were recruited and consented. The consented participants were 50 (SD 15) years old. Most of these individuals identified as female at birth (n=596, 78.84%) and White (n=662, 85.75%). Other racial groups included African American (n=55, 7.12%); Asian (n=26, 3.37%); Hawaiian, Pacific Islander, or Native American (n=12, 1.55%); and others (n=17, 2.20%). Further, 6.87% (n=52) of the consented participants across all races identified as Hispanics. With regards to income, 1.17% (n=8) were within the lowest range of income (<US $10,000), 4.53% (n=31) earned between $10,000 and $29,999, 7.44% (n=51) earned between $30,000 and $49,000, 13.14% (n=90) earned between $50,000 and $69,999, 16.06% (n=110) earned between $70,000 and $99,999, 26.13% (n=179) earned between $100,000 and $149,9999, and 31.53% (n=216) earned more than $150,000. With regards to education, 0.41% (n=3) had no school education, 4.75% (n=35) had a basic education from elementary school up to high school diploma or alternative credentials, 22.66% (n=167) had varying levels of college education up to bachelor’s degree, 71.77% (529) had higher education attainment, and 0.41% (n=3) did not respond.

## Discussion

### Principal Findings

The preliminary findings of COVIDsmart demonstrate the ability to remotely recruit and engage participants for ongoing data collection and research opportunities of the COVID-19 pandemic in Virginia. Our findings further inform preferred recruitment strategies for success across diverse communities and participants' interest in remote digital health studies.

Although COVID-19 is causing a pandemic worldwide, it is also favoring the rapid adoption of digital solutions and advanced technology tools in health care. With the massive number of research studies conducted on the COVID-19 epidemiology, diagnosis, and management, the time and resources required to identify collaborators, the right tools, and determining measures can cause teams to move ahead quickly at the risk of losing scientific rigor. Over 250,000 publications have been created from research studies focused on COVID-19 related to vaccines, prevention, and biotechnology [12]. COVIDsmart used digital tools to evaluate individual study participants along with the holistic impact of COVID-19 on communities. Impacts on health (social, mental, and physical) were collected remotely, including mood, social communication and isolation, alcohol dependency, sleep deprivation, and other behavioral risk indicators over time.

Virginia’s sociodemographic distribution offers a unique and important source to study the multifaceted impact of the COVID-19 pandemic in a broad range of lifestyle, environmental, economic, and social settings. COVIDsmart project management was, therefore, primarily focused on creating a digital cohort that represented Virginia’s diverse demographic characteristics. In a multi-institutional effort, COVIDsmart took a diversity-focused digital recruitment approach that was integrated with remote collection of formative surveys to gain a better understanding of participants’ experiences and successful recruitment strategies across the state. While we were able to recruit and consent participants across a broad range of demographic groups, the current results also indicate the underrepresentation of non-White racial or ethnic groups, males, younger adults, and individuals with lowest range of income and education. Our future endeavors will, therefore, focus on recruiting more participants from these groups.

The DHSP allowed COVIDsmart to access large, diverse participant groups remotely across the state without requiring in-person study-related visits. Continued use of the secure, validated DHSP platform in future studies could greatly scale and manage study enrollment, participation, and retention [31,32]. The use of such a platform allowed the flexibility in study implementation as the research team modified the study approach to account for ever-changing information on COVID-19.

Preliminary findings have shown the overall relationship of effective participation and that the reasons to participate were due to altruism. This also indicates that incentives may not be the primary motivation for recruitment. Since the study is ongoing, retention outcomes are not presented here and will be reported upon study completion. The study participants are described through the initial consent and set of baseline surveys which may reveal the extent of the recruitment materials and the study understanding alignment or misalignment.

### Limitations

This paper describes lessons around collaboratively deploying a digital research study using digital recruitment and remote participant enrollment solely through publications, digital advertisements, and social media. This study demonstrates the ability to effectively measure study convergence rates by categorization and tracking of digital recruitment methods.

The findings presented in this paper could be strengthened by a larger sample size to further assess the achievement of diversity by recruitment methods such as representation in age, income, ethnicity and race, and method consideration for word-of-mouth digital advocacy.

Another limitation is that this study was launched in March of 2021, a year after the World Health Organization declared the COVID-19 outbreak a pandemic. Therefore, COVIDsmart did not capture the short-term postpandemic impact. This may result in an incomplete capturing of the immediate physical and psychological effects of the pandemic on Virginian residents. Due to the timing of the study, data collected may be affected by COVID-19 fatigue, where the overall messaging may be tiring and overused, as participants have been through so much, and advertisements and questions are no longer meaningful. The timing of study initiation may also affect retention rates, as participants may have signed up and withdrawn immediately. Despite the study producing an effective yield of participants, we cannot rule out the potential risk of bias. On reflection, we recognize that being more adaptive while releasing 1 survey instrument and growing the cohort from the start of the pandemic would have produced more participant recruitment and satisfied varying appetites of participants to contribute to the research.

As this study was entirely remote, it required internet access. Due to digital inequities across low-income or rural areas with no internet access or low bandwidth, this may have limited the study inclusion. Finally, a lack of evaluation of our coproduction process limits the ability to draw conclusions on the long-term impact of COVIDsmart study processes. Future research should include an evaluation of the coproduction processes to assess the partnership and the impact of coproduction on the success of the research study.

### Conclusion

The COVID-19 pandemic has accelerated the need for digital transformation in research. COVIDsmart is a statewide prospective cohort to study the social, physical, and mental health impacts of COVID-19 on the diverse population of Virginia. The study design, project management, and collaborative efforts led to the development of digital recruitment, enrollment, and comprehensive survey instruments to collect general health, COVID-19 exposure and vaccinations, mood and behavioral disorders, lifestyle, and socioeconomic data. Preliminary findings may inform successful recruitment techniques across diverse communities and participants' interest for remote digital health studies in the future.

## Abbreviations

DHSP: digital health solutions platform
EVMS: Eastern Virginia Medical School
HADSI: Healthcare Analytics and Delivery Science Institute
HIPAA: Health Insurance Portability and Accountability Act
